# Gluconeogenesis in Plants: A Key Interface between Organic Acid/Amino Acid/Lipid and Sugar Metabolism

**DOI:** 10.3390/molecules26175129

**Published:** 2021-08-24

**Authors:** Robert P. Walker, Zhi-Hui Chen, Franco Famiani

**Affiliations:** 1 Independent Researcher, Lancashire, Bolton BL2 3BG, UK; 2 School of Life Science, University of Dundee, Dundee DD1 5EH, UK; 3 Dipartimento di Scienze Agrarie, Alimentari e Ambientali, Università degli Studi di Perugia, 06123 Perugia, Italy

**Keywords:** gluconeogenesis, malate, malic enzyme, nitrogen metabolism, organic acids, phosphoenolpyruvate carboxykinase, pyruvate orthophosphate dikinase, vacuole

## Abstract

Gluconeogenesis is a key interface between organic acid/amino acid/lipid and sugar metabolism. The aims of this article are four-fold. First, to provide a concise overview of plant gluconeogenesis. Second, to emphasise the widespread occurrence of gluconeogenesis and its utilisation in diverse processes. Third, to stress the importance of the vacuolar storage and release of Krebs cycle acids/nitrogenous compounds, and of the role of gluconeogenesis and malic enzyme in this process. Fourth, to outline the contribution of fine control of enzyme activity to the coordinate-regulation of gluconeogenesis and malate metabolism, and the importance of cytosolic pH in this.

## 1. Introduction

**Figure 1:**
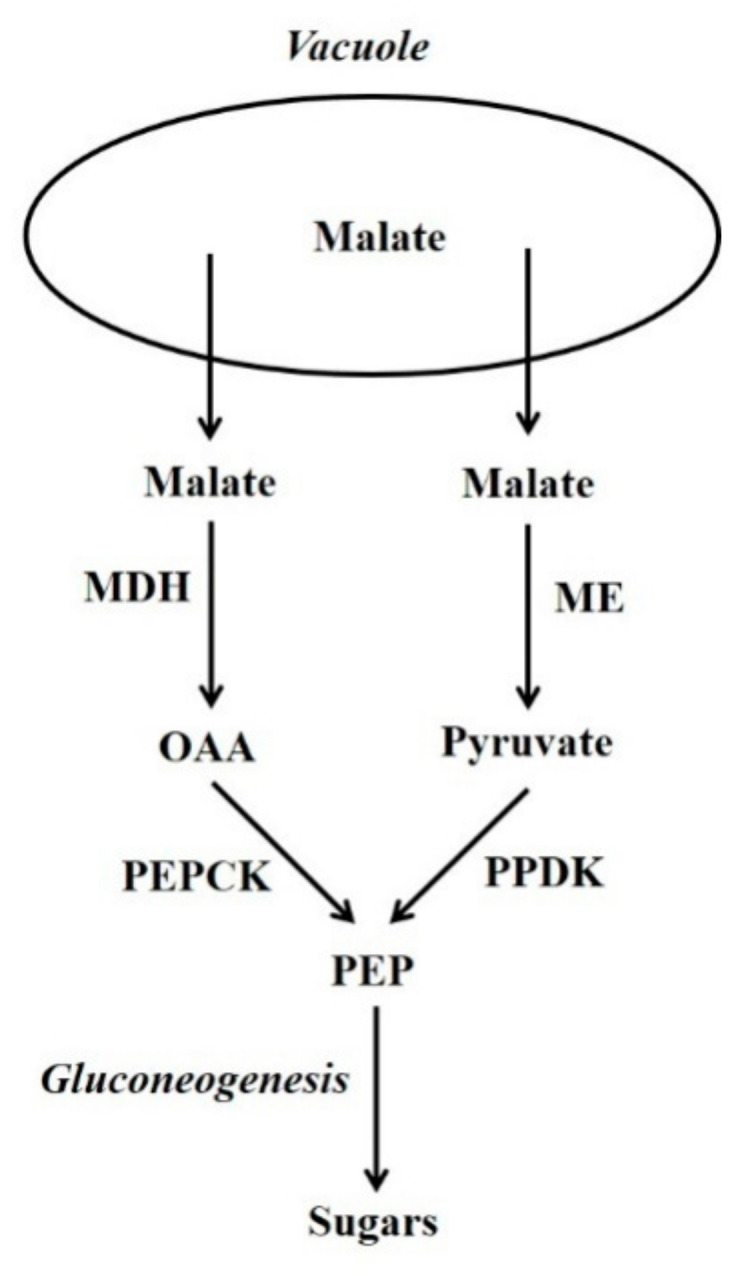
The PEPCK and PPDK gluconeogenesis pathways. OAA = oxaloacetate; PEP = phosphoenolpyruvate; MDH = malate dehydrogenase; ME = malic enzyme; PEPCK = phosphoenolpyruvate carboxykinase; PPDK = pyruvate orthophosphate dikinase.

In plants, certain non-carbohydrate compounds such as lipids, amino acid carbon skeletons, and Krebs cycle organic acids (i.e., malic and other acids that are intermediates of the Krebs cycle) can be converted to sugars by a process called gluconeogenesis. Lipids and many amino acids are first converted to Krebs cycle organic acids, and the latter are then converted to sugars by gluconeogenesis. Thus, gluconeogenesis is a key interface between organic acid/amino acid/lipid metabolism and sugar metabolism. Two pathways can be used by plant gluconeogenesis [1,2,3]. One pathway employs phosphoenolpyruvate carboxykinase (PEPCK; oxalacetate (OAA) + ATP 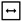 phosphoenolpyruvate (PEP) + CO_2_ + ADP) in conjunction with malate dehydrogenase (MDH), whilst the alternate pathway employs pyruvate orthophosphate dikinase (PPDK; ATP + pyruvate + Pi 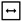 AMP + PEP + PPi) in conjunction with malic enzyme (ME) (Figure 1) [1,2,4]. Although the presence of PEPCK protein is constitutive in some plant tissues (e.g. the flesh of fruits at certain stages of development and the leaves of many C_4_ and Crassulacean acid metabolism [CAM] plants) in others it is not; nevertheless, in many of the latter its presence can be induced by certain stimuli. The situation is similar for PPDK, and for both enzymes their presence confers on most tissues the capacity for gluconeogenesis [2,3]. It should be noted that, in the photosynthetic tissues of C_4_ leaves, both PEPCK and PPDK function in the C_4_ photosynthesis and not gluconeogenesis [2,3]. By contrast, MDH and ME are present in numerous tissues in which they do not function in gluconeogenesis [2,3]. Whether the PEPCK or PPDK pathway is utilised, is dependent on the species, tissue, developmental stage and potentially environmental factors such as N-supply.

The vacuolar storage, subsequent release and metabolism of certain Krebs cycle acids (e.g., malic and citric) and nitrogenous compounds (e.g., ammonium and alanine) is of widespread occurrence in plants: and of fundamental importance when considering both gluconeogenesis [5,6,7], and certain other aspects of plant metabolism [8,9]. The vacuole occupies a considerable proportion of the volume of most plant cells, and this is one reason why it is well suited to the storage of these compounds. In addition, enzymes that metabolise malate and nitrogenous compounds are generally not present in the vacuole [7,10]. The storage-release of these metabolites can be associated with various processes such as osmoregulation, coordination of the import and utilisation of nitrogenous compounds, regulation of metabolite concentrations in different subcellular compartments and CAM. In association with gluconeogenesis this vacuolar storage-release is likely to occur in many plant organs/tissues (e.g., flowers, seeds, fruits, leaves, roots, vasculature, stomata and trichomes), and be involved in diverse processes such as vascular function, fruit and seed development, seed germination, osmoregulation, nitrogen metabolism, defence and responses to various stresses [7,11,12,13,14,15,16].

**Figure 2:**
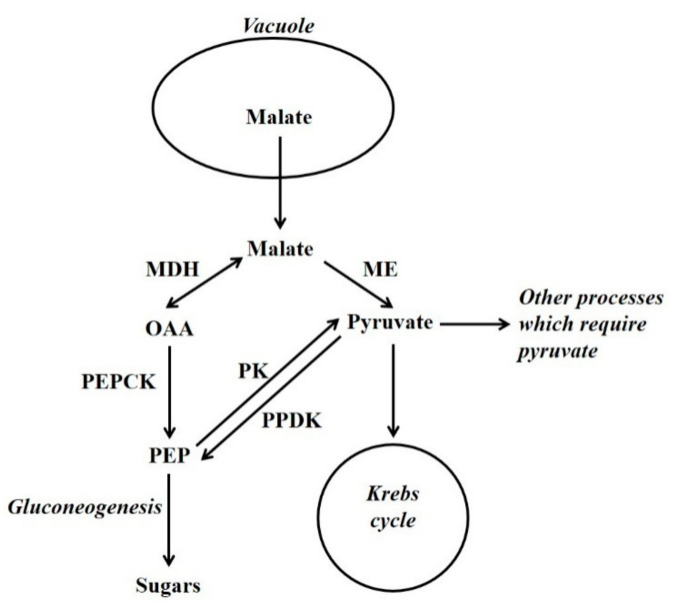
The utilisation of vacuolar-released malate by gluconeogenesis and other processes such as the Krebs cycle. OAA = oxaloacetate; PEP = phosphoenolpyruvate; MDH = malate dehydrogenase; ME = malic enzyme; PEPCK = phosphoenolpyruvate carboxykinase; PK = pyruvate kinase; PPDK = pyruvate orthophosphate dikinase.

In many tissues gluconeogenesis is associated with the metabolism of Krebs cycle acids and/or nitrogenous compounds when they are released from the vacuole. These compounds, or their breakdown products, are used by processes such as the Krebs cycle. Gluconeogenesis occurs when the demand of other processes is exceeded (Figure 2) [7,16]. Thus, at these times the amount of these compounds released from the vacuole is such that glycolytic flux is not necessary to provide metabolic intermediates. The allocation of vacuolar-released Krebs cycle acids between gluconeogenesis and other processes must be regulated. There is evidence that in a range of tissues (e.g., leaves of both C_3_ and CAM plants, fruit flesh and stomata), there are times when Krebs cycle acids are synthesised and deposited in the vacuole, and times when they are released from the vacuole and metabolised [2,7,16,17,18]. In leaves of C_3_ plants, it appears that this release occurs largely during the nocturnal period [18,19]. Indeed, in these leaves modelling and NMR studies have shown that much of the carbon used in the synthesis of glutamate is derived from CO_2_ fixed in a previous nocturnal period, and stored in the vacuole as citrate [18,20,21]. Clearly, Krebs cycle acids are often synthesised from sugars, and a key enzyme utilised in this is phophoenolpyruvate carboxylase (PEPC; PEP + HCO_3_^−^ → OAA + Pi) [2]. PEPC together with enzymes used in malate breakdown, such as malate dehydrogenase (MDH), PEPCK and cytosolic NADP-malic enzyme (NADP-ME), are present in the cytosol of the same cells [2,22]. Hence, there must be mechanisms that coordinate flux through malate synthetic and degradative enzymes.

Malate serves an anaplerotic role in replenishing Krebs cycle intermediates. This is necessary because intermediates are withdrawn from the cycle, and used in biosynthesis (e.g., amino acids) or storage (e.g., citrate). When malate is released from the vacuole it can fulfil this anaplerotic requirement. However, for the Krebs cycle to function primarily in the generation of NADH/ATP (i.e., intermediates are not withdrawn from the cycle) pyruvate is required. This is because of the stoichiometry of the Krebs cycle: if malate/OAA (4C) enter the cycle they become citrate (6C) (in this situation pyruvate is also required and this forms acetyl CoA, that is utilised together with OAA, by citrate synthase, to form citrate). Citrate then flows through the cycle to form malate and 2C are lost as CO_2_. Thus, in this situation 6C have entered the cycle and only 2C have left it [2]. Thus, for malate to be fully oxidised it must first be converted to pyruvate (to prevent a build-up of intermediates of the cycle). This conversion of malate to pyruvate is an example of cataplerosis, and potentially both PEPCK (in conjunction with pyruvate kinase [PK]) and ME can function as cataplerotic enzymes [2,23] (Figure 2). The importance of a supply of pyruvate to the mitochondrion is illustrated by a recent study [24]. Gluconeogenesis can also occur from amino acid carbon skeletons [7,25,26,27,28]. For example, in maize endosperm there is evidence that vacuolar release of alanine results in gluconeogenesis via PPDK [7]. The process of storage, release and subsequent metabolism of Krebs cycle acids and the association with nitrogen metabolism has likely provided building blocks for the evolution of processes such as CAM, stomatal metabolism, C_4_ photosynthesis and the biochemical pH stat [7,18].

## 2. Potential Occurrence of Gluconeogenesis

### 2.1. Germinating Seeds

In germinating seeds, stored lipids and a proportion of stored proteins are converted to sugars by gluconeogenesis [25,29,30,31,32]. Malate is produced from lipids by the glyoxylate cycle [30,33,34]. It is possible, in order to assist in coordinating lipid/protein breakdown with the utilisation of these breakdown products, that a proportion of this malate is stored temporarily in the vacuole. Until recently it was thought that only the PEPCK gluconeogenic pathway was utilised in germinating seeds, however, recent work indicated that the PPDK pathway contributes in Arabidopsis [4]. Nevertheless, in cotyledons from germinating cucumber PEPCK was very abundant, whereas PPDK was not detected (our unpublished data), and this suggests that PPDK makes little or no contribution to gluconeogenesis in this organ. Clearly, in order to evaluate how widespread is the use of the PPDK pathway, the abundance of PEPCK and PPDK (protein/activity) in germinating seeds of a range of species needs to be determined.

### 2.2. Developing Seeds

Radiolabelling studies have shown that gluconeogenesis from amino acids occurs in developing seeds [7,26,27,28,35]. Although the glyoxylate cycle is active in many developing seeds, it is often not linked to gluconeogenesis [36]. PEPCK and PPDK are often present in developing seeds, however, their abundance depends on the species, stage of development and tissue [7,37,38,39]. In maize kernels PPDK is present in some tissues, such as the endosperm, and PEPCK in others, and both enzymes appear to have a role in gluconeogenesis associated with nitrogen metabolism [7]. In developing cherry and plum seeds PEPCK is abundant, whereas PPDK was not detected [40,41]. In the seeds of several species the abundance of PEPCK is correlated with the deposition of storage proteins, as is PPDK in maize endosperm [7,38,42]. The abundance of PEPCK in developing seeds can be increased greatly by either feeding seeds certain nitrogenous compounds in vitro, or by increasing the amount of nitrogenous compounds fed to the plant [38,42].

### 2.3. Senescing Tissues

The glyoxylate cycle occurs in both senescing and carbon-starved tissues, and it has been proposed to be linked to gluconeogenesis. However, this is unlikely, and one role may be in the anaplerotic replenishment of the Krebs cycle [43,44]. PPDK has been proposed to function in amino acid metabolism, associated with the export of nitrogenous compounds out of senescing leaves [45]. However, this role might not be associated with senescence per se, because PPDK protein is present in mature tomato leaves and its abundance g^−1^ FW does not increase during senescence [7].

### 2.4. Vasculature

PEPCK is present in the vasculature of a range of tissues, and this abundance is often increased by feeding the plant ammonium but not nitrate [46,47]. In the vasculature of rice leaves there is evidence that PEPCK plays a role in metabolism associated with xylem-phloem transfer of nitrogenous compounds [48]. In leaf vasculature PPDK functions in nitrogen metabolism [45], and gluconeogenesis from alanine occurs in cottonwood leaves [49]. Thus, it is possible that in leaves PPDK could also function in nitrogen metabolism that is associated with xylem-phloem transfer of nitrogenous compounds.

### 2.5. Roots

In roots PEPCK appears in response to feeding them ammonium, and is localised in the pericycle and vascular tissues. PPDK appears in roots subjected to anaerobic conditions [7,46,47,50]. Some studies have shown that PEPCK abundance in roots is not increased by anaerobic conditions, whilst others have, and reasons for these differences between studies need to be investigated [7,47,51].

### 2.6. Trichomes and Plant Defense

PEPCK is often present in defence tissues, such as some trichomes and the extrafascicular phloem of Curcurbits, in tissues undergoing lignification and in tissues challenged by pathogens. In many of these tissues large amounts of ammonium are likely to be produced by phenylalanine ammonia lyase (PAL) during the synthesis of phenolics [40,41,43,47,52,53,54,55]. Thus, gluconeogenesis could be used in the metabolism of malate released from the vacuole which is associated with vacuolar storage/release of ammonium. Further, it is possible that gluconeogenesis could be associated with osmoregulation. Thus, in trichomes and certain other tissues, both malate and sugars might be used to control processes such as expansion and import of materials [13,56,57].

### 2.7. CAM Plants

In photosynthetic tissues of CAM plants, malate/citrate is synthesised at night and stored in the vacuole. During the day, these acids are released from the vacuole, decarboxylated by PEPCK or ME and the released CO_2_ is used by photosynthesis. Pyruvate produced by ME is converted to PEP by PPDK. PEP is also produced from malate by malate dehydrogenase (MDH) in conjunction with PEPCK. PEP is then converted to sugars by gluconeogenesis [1,2].

### 2.8. Stomata

Malate/citrate and sugars are abundant osmotica in stomata, and both can be important in turgor regulation associated with stomatal opening/closing [58,59,60,61,62]. Both PEPCK and PPDK could potentially play a role in converting malate/citrate, which is released from the vacuole, to sugars [15,17,58,60]. However, there is evidence that at least in certain species PEPCK may be more important [58]. PEPCK has been localised in motor cells of rice leaves [63], and in these a similar role in osmoregulation is possible.

### 2.9. PPDK and Gluconeogenesis in Fruits

PPDK protein was not detected in the flesh of a range of soft fruits, plum, cherry or grape [22,41,64,65]. PPDK protein was detected in tomato flesh, and in this its abundance corresponded to an estimated activity of c0.003–0.014 µmol min^−1^ g^−1^ FW [12]. PEPCK activity in tomato, grape and cherry flesh is c0.1–0.3 µmol min^−1^ g^−1^ FW [16,64,66]. PPDK polypeptide has been detected in peach flesh [67,68]. However, throughout the ripening of peach flesh the amount of PPDK polypeptide is very low and its estimated activity is around 0.0003–0.0014 µmol min^−1^ g^−1^ FW [12]. By contrast PEPCK is quite abundant in peach flesh throughout ripening (c0.18 U g^−1^ FW), and this suggests that the bulk of any gluconeogenic flux utilises PEPCK [12]. Similarly, if PPDK is present in grape pericarp under normal conditions of growth it is likely to be at very low abundance compared to PEPCK [52]. Nevertheless, it is possible that the abundance of PPDK increases under certain conditions (e.g., low O_2_) [69]. Therefore, in the flesh of most fruits it appears that the PEPCK pathway makes the largest contribution to gluconeogenesis [12]. PPDK is abundant in the peel of cactus pear fruits, however, in these it is a component of CAM [70]. Substantial amounts of PPDK activity were detected in extracts of bean fruit (*Phaseolus vulgaris* pod) [71].

### 2.10. Glyoxylate Cycle and Gluconeogenesis in Fruits

Isocitrate lyase (ICL) and malate synthase (MS) are key enzymes of the glyoxylate cycle. In the flesh of ripening banana fruits both MS transcripts and ICL activity are present [72,73]. ICL activity has been measured in extracts of cucumber flesh [74], and there is a very low expression of a MS gene in this tissue [75]. In extracts of pumpkin flesh, from fruits at the time of commercial harvest, ICL polypeptide and activity were not detected, whereas, in slices of the fruits incubated under darkness they were [76]. Nevertheless, the activity of ICL is very low in the fruits in which it has been detected: in ripening banana flesh (c0.0001 µmol min^−1^ g^−1^ FW) [73] and in cucumber flesh (c0.008 µmol min^−1^ g^−1^ FW) [74]. ICL polypeptide was either absent or at very low abundance in the flesh of both grape and some soft fruits [22,65]. Therefore, in the flesh of most fruits under normal conditions of growth it appears that the glyoxylate cycle makes little or no contribution to providing substrate for gluconeogenesis [12].

### 2.11. PEPCK and Gluconeogenesis in Fruits

Radiolabelling studies in the 1960s provided evidence for the occurrence of gluconeogenesis from malate in the flesh of grape berries [77,78], and it was hypothesised that the PEPCK pathway was used [79]. A detailed study established the presence of PEPCK in grape berries [80]. Subsequently, radiolabelling studies demonstrated gluconeogenesis from malate in the flesh of both ripening tomato and cherry fruits [81,82,83]. Studies in transgenic tomato fruits containing altered amounts of PEPCK support the view that the enzyme can participate in gluconeogenesis [84,85,86]. The presence of PEPCK in the flesh of a range of other fruits suggests that gluconeogenesis can occur in these [11,22,41,64,87]. In the flesh of fruits, gluconeogenesis and certain other aspects of malate metabolism are not thought to be associated with photosynthesis [88]. Consistent with this there is no correlation between the abundance of Calvin cycle enzymes and PEPCK in either the flesh or endocarp of several fruits [22,41,87].

## 3. Gluconeogenesis and the Vacuolar Release of Malate/Citrate and Nitrogenous Compounds

### 3.1. VacuolarMalate/Citrate Storage and Release

The bulk of the malate content of fruit flesh is located in the vacuole [10]. In grape pericarp it was proposed that during ripening malate was released from the vacuole, and its metabolism by NADP-ME provided pyruvate for the Krebs cycle, whereas metabolism by PEPCK produced PEP that was used in gluconeogenesis [89]. The view developed that in ripening grape pericarp glycolysis was largely inhibited, and the predominant substrate used by metabolism was malate [89,90,91]. However, the latter was shown to be incorrect, and if the whole ripening period was considered sugars provided the bulk of metabolic substrate [5,92]. In the ripening flesh of other fruits, sugars are also likely to provide the bulk of the metabolic substrate [11,93]. In the fruits of many species, PEPCK is present when there is no net decrease in their Krebs cycle acid content [93]. Further, in grape pericarp radiolabelling studies have shown that malate can be converted to sugars before ripening, and at this time the malate content increases [94]. These studies raised the question as to why gluconeogenesis occurred. The most likely explanation is that there is a turnover of the vacuolar malate/citrate pool throughout development [11,16]. Thus, at certain times malate/citrate is released from the vacuole, and when their breakdown products exceed the demands of other processes, gluconeogenesis occurs. Clearly maintaining an appropriate concentration of malate in the extravacuolar compartment is crucial because perturbations in this affect various processes such as starch metabolism [95]. Then, sometime later malate efflux stops, and malate is resynthesised and transported into the vacuole [11,16]. These malate effluxes are likely to be associated with nitrogen metabolism (see below) and/or osmoregulation. Osmoregulation plays an important role in the pericarp of fruits; and is involved in fruit softening, cell expansion and the import of materials [14,57,96].

### 3.2. Ammonium Metabolism

In a number of tissues PEPCK is associated with ammonium metabolism [2,38,42,48,52,97,98,99,100,101]. For example, PEPCK protein and activity are increased greatly in a range of plant tissues such as: roots, vasculature and developing seeds when they are fed nitrogenous compounds such as ammonium or asparagine (ammonium is often released from asparagine when it is metabolised) [38,42,46,47,52]. Ammonium can accumulate in both the flesh of fruits and other tissues such as roots and developing seeds [11,40,42,46,47,64,102,103]. The bulk of the intracellular contents of both malate and ammonium are located in the vacuole [104]. In sinks, ammonium can arise from its import in the xylem, its synthesis from imported nitrate and from the metabolism of amino acids and amides by asparaginase or glutamate dehydrogenase. In addition, ammonium is produced during the synthesis of many phenolic compounds as a result of PAL [105].

In leaves the release of malate/citrate and ammonium from vacuoles, and their incorporation into amino acids, is coordinated and occurs at certain times during the diurnal cycle [9,106]. In other tissues, such as the flesh of fruits and developing seeds, vacuolar release of malate/citrate and ammonium and their assimilation into amino acids are also likely to be coordinated. In leaves malate/citrate metabolism is intimately linked with pH regulation that is associated with nitrogen metabolism [9,106]. In sink tissues, pH regulation is also involved in the utilisation of malate/citrate in ammonium assimilation [11,97]. For example, protons are consumed when ammonium, produced by the metabolism of glutamine or asparagine, is stored in the vacuole and the carbon skeletons of these amides are metabolised. If malic/citric acid is synthesised in the cytosol, and malate/citrate transported into the vacuole this will counteract an increase in cytosolic pH. Subsequently, when ammonium is released from the vacuole and assimilated into amino acids, protons will be produced if sugars provide the amino acid carbon skeletons. However, if malate/citrate is released from the vacuole and used together with sugars to produce these carbon skeletons no protons are produced [97].

**Figure 3:**
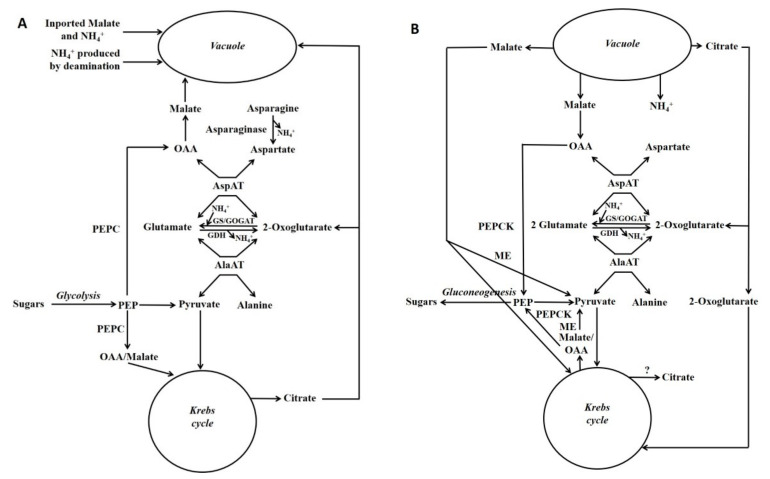
Gluconeogenesis from malate and its association with nitrogen metabolism. Simplified scheme depicting gluconeogenesis associated with the metabolism of asparagine or ammonium: storage phase of these metabolites (**A**); utilisation phase (**B**) (Reproduced from Walker et al. [7]). Fluxes through different reactions in the schemes will differ according to factors such as which nitrogenous compound (e.g., asparagine [e.g., maize pedicel], glutamine or ammonium [e.g., maize root fed ammonium]) is the major input. Glutamate, aspartate, pyruvate and PEP are the precursors of most amino acids [105], and in both situations these metabolites could be produced. GDH = glutamate dehydrogenase; GOGAT = glutamine oxoglutarate aminotransferase; GS = glutamine synthase; ME = malic enzyme; OAA = oxalacetate; PEP = phosphoenolpyruvate; PEPC = phosphoenolpyruvate carboxylase.

**Figure 4:**
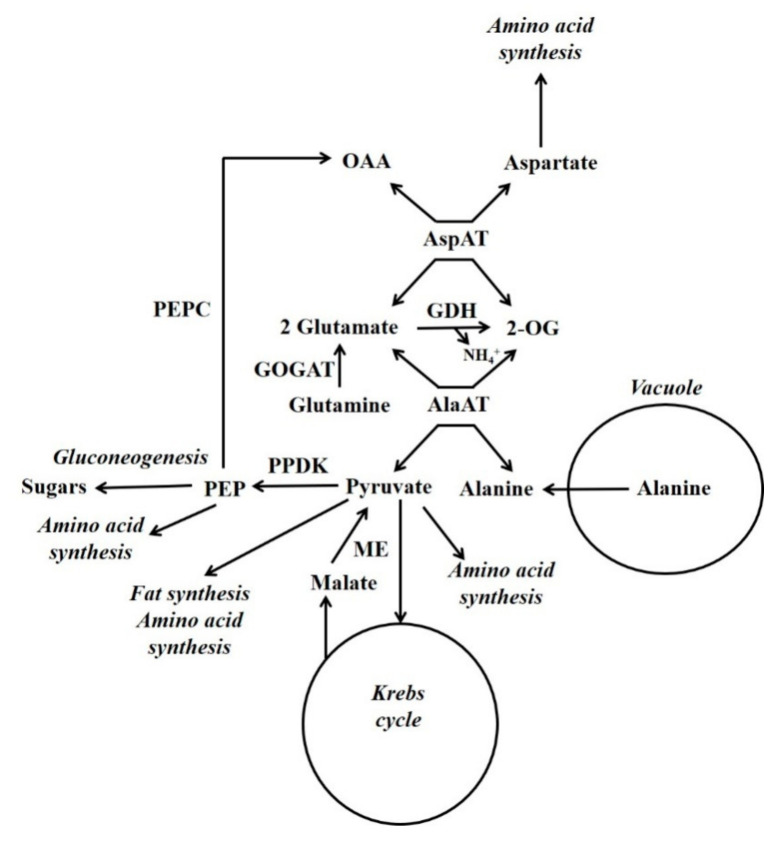
Gluconeogenesis from vacuolar-released alanine. Simplified scheme outlining the functions of PPDK in gluconeogenesis and nitrogen metabolism (Reproduced from Walker et al. [7]). GDH = glutamate dehydrogenase; GOGAT = glutamine oxoglutarate aminotransferase; ME = malic enzyme; OAA = oxaloacetate; 2-OG = 2-oxoglutarate; PEP = phosphoenolpyruvate; PEPC = phosphoenolpyruvate carboxylase.

Schemes depicting events occurring during either the accumulation of malate/citrate in the vacuole or during their subsequent release are shown in Figure 3. Glutamate dehydrogenase (GDH) is considered to play an important role in regulating the cytoplasmic glutamate concentration [107]. Glutamate concentration is linked to the concentration of both aspartate and alanine because of the AspAT and AlaAT reactions. Thus, adjusting glutamate concentration by GDH can also potentially alter aspartate and alanine concentrations. For example, in the case of vacuolar efflux of alanine, GDH allows alanine to be metabolised without the requirement to synthesise large amounts of 2-OG (Figure 3 and Figure 4).

### 3.3. Alanine Metabolism

In developing maize endosperm radiolabelling has shown that there can be a massive gluconeogenic flux from alanine and glutamine [26,27] (Figure 4). In this tissue PPDK and not PEPCK is abundant, and alanine can account for a considerable proportion of the amino acid content [7]. In some plant tissues alanine is stored in the vacuole [108]. When alanine is released from the vacuole it is metabolised by cytosolic alanine aminotransferase (AlaAT) (alanine + 2-OG 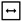 glutamate + pyruvate). It is hypothesised that gluconeogenesis occurs when the amount of pyruvate produced by cytosolic AlaAT is in excess of the requirements of other processes [7]. Under hypoxia, roots accumulate alanine and PPDK abundance increases massively [7,50]. This accumulation of alanine allows the production of ATP under low O_2_ conditions, and involves a reconfiguration of central metabolism which is outlined by Rocha et al. and António et al. [109,110]. Alanine content decreases when O_2_ is supplied [111], and it is possible that one function of PPDK could be in the metabolism of this alanine in a similar way to that suggested for developing maize endosperm [7].

## 4. Plant PEPCK Regulation and PEPCK Genes

**Figure 5:**
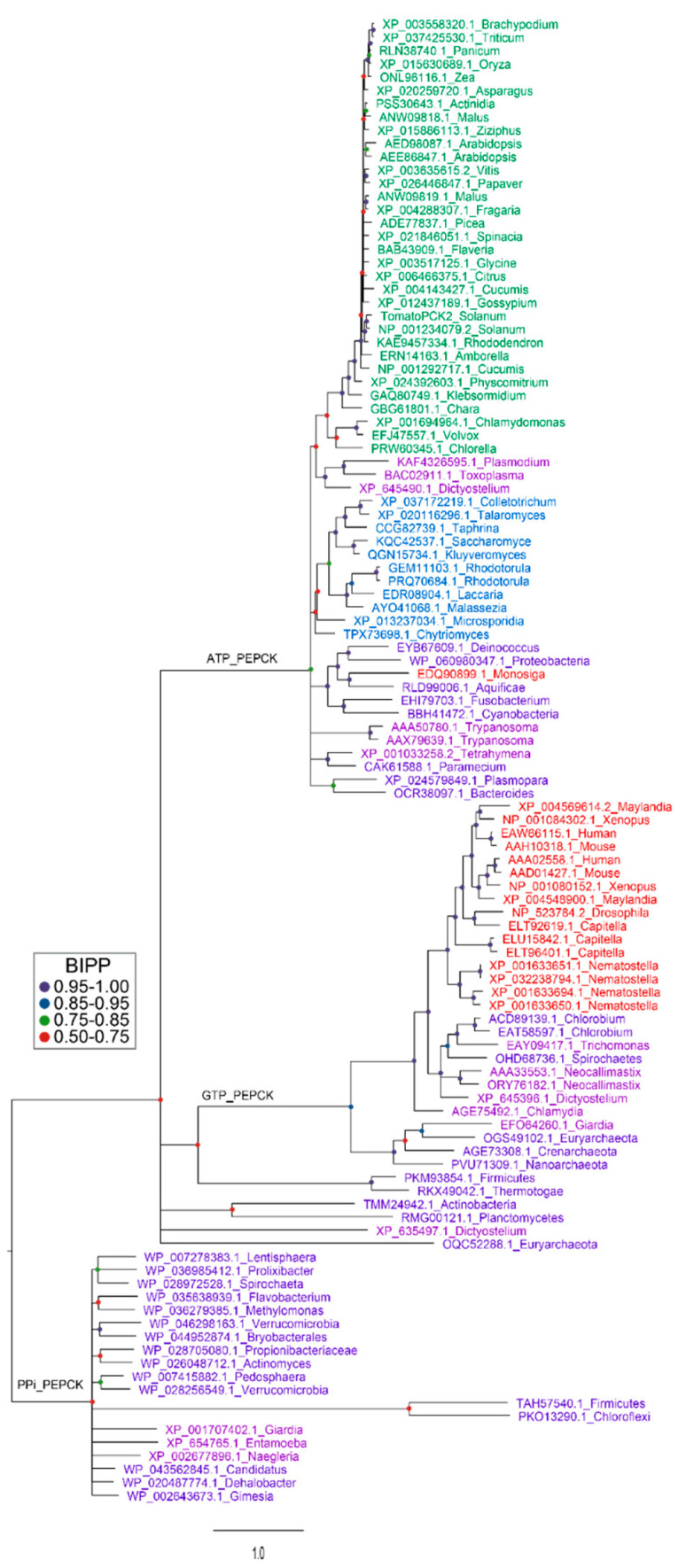
Phylogenetic analysis of PEPCK proteins from three domains of life. Several representative PEPCK protein sequences were selected for each phylum of three domains of life [130] including bacteria [131,132], protozoans and fungi [133], plants [134] and metazoans in the NCBI database. Protein sequences were aligned using Clustal W [135]. After deletion of segments with poor consensus alignment, sequences were subjected to Bayesian inference for establishment of phylogenetic relationships between proteins [136]. Analysis were run for 5 million generations under a mixed amino-acid model with rate variation between sites estimated by a gamma distribution. Bayesian inference posterior probabilities (BIPPs) of tree nodes are indicated by coloured dots. Gene identifiers of the proteins are color-coded to represent the phyla from which they are derived. Green for plants, blue for fungi, pink for protozoans, purple for bacteria and red for metazoans. Corresponding species names are listed by the side of accession number on each branch of the tree. Note for the plant species there are very little differences in the amino acid sequence of the protein apart from in the c12 kD N-terminal extension. Thus, the reconstructed phylogeny of the plant enzyme is based largely on this part of the protein.

**Figure 6:**
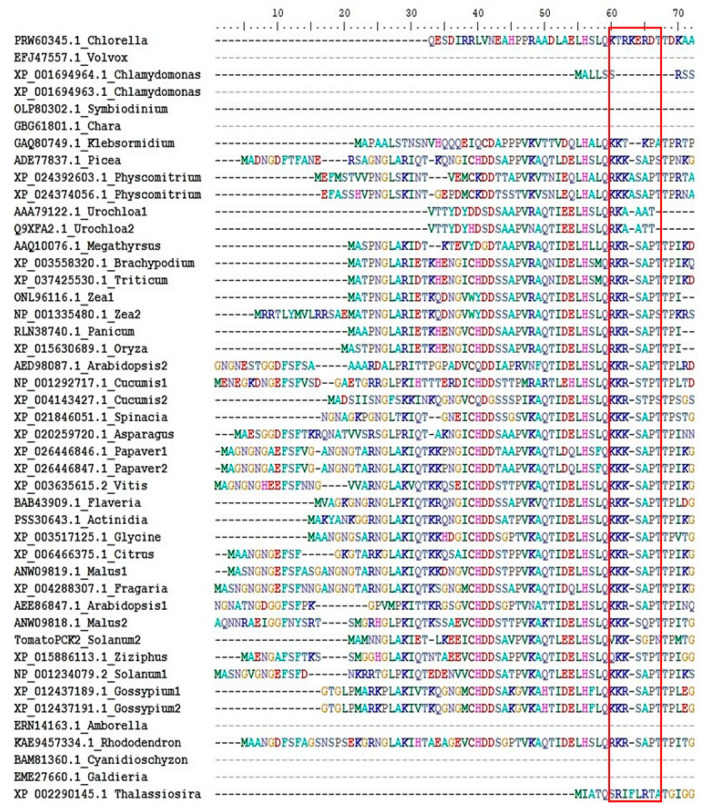
Alignment of N-terminal extension sequences of plant PEPCKs. Several representative PEPCK protein sequences, from the phylum of plants and algae [134], were selected from the NCBI database. Protein sequences were aligned using Clustal W [135]. The alignment of N-terminal extension sequences was edited and extracted using the BioEdit programme. Species names are listed by the side of accession number. The phosphorylation motif (KK/RXSXPT) or its absence is shown in the red box.

There are three forms of PEPCK: PEPCK-ATP, PEPCK-GTP and PEPCK-PPi, however, plants only contain PEPCK-ATP [97,112]. These forms likely arose from a common ancestor, [97,112] (Figure 5). In plants there is often more than one form of a given enzyme. For example, there are different forms of malic enzyme, and each of these forms shows a higher sequence similarity to the same form from other plants species than to the other forms from the same species [113,114]. This is not the case for PEPCK (at least for the forms not involved in C_4_ photosynthesis), and if a plant contains more than one PEPCK gene, these genes are usually more closely related to each other than to PEPCK genes from other plants (Figure 5). A characteristic feature of PEPCK from both plants (angiosperms, gymnosperms and bryophytes) and green algae (from which plants evolved) is the possession of an N-terminal extension [31,115,116] (Figure 6). Although a number of amino acid residues that comprise plant PEPCK are phosphorylated [117], only one site (cAMP-dependent protein kinase site) is known to be subject to reversible phosphorylation [2,97,118,119]. In maize leaves, although this site is phosphorylated [117], it is not subject to large changes in phosphorylation status during the diurnal cycle [115,119]. This site is located within the N-terminal extension, and changes in its phosphorylation status alter the catalytic properties of the enzyme [119,120]. This phosphorylation site is present in the enzyme from mosses, gymnosperms and angiosperms but not green algae (Figure 6). Phosphorylation of PEPCK lowers its affinity for its substrate OAA, and this effect is dependent on both ATP/ADP ratio and concentrations of magnesium and manganese [97,119,121]. All three types of PEPCK are not generally thought to be modulated by most other metabolites if in vitro assay conditions approximate those within the plant cytosol [97,121]. However, more recent work reported that PEPCK activity is modulated by a plethora of metabolites [122,123]. Clearly this controversy needs to be resolved. The N-terminal extension of PEPCK is susceptible to proteolysis upon extraction of the tissue [31]. It has been suggested that this could be a mechanism used to modulate the activity of the enzyme in vivo [123]. However, such a mechanism is unlikely to contribute to the regulation of PEPCK in at least some plant tissues such as leaves of both CAM and C_4_ plants: because little or no cleavage of the enzyme was noted in leaves harvested from plants during either the night or day [118].

## 5. Coordinate Regulation of Malate Metabolism and Gluconeogenesis

Enzymes involved in malate synthesis such as PEPC and MDH, and those involved in its degradation (e.g., MDH and PEPCK and cytosolic NADP-ME), are present in the cytosol. Thus, regulatory mechanisms are required to activate malate-synthetic enzymes and inactivate malate-degradation enzymes when malate is accumulated in the vacuole, and the reverse when malate is released from the vacuole [2]. The coordination of PEPCK and PEPC activities involves phosphorylation of both enzymes in conjunction with changes in metabolite concentration and pH [2,97,118,119,124,125]. Trehalose 6-P is a key regulatory metabolite that is involved in coordinating changes in flux between sucrose and organic acid/amino acid synthesis [19]. Clearly this metabolite is likely to play a role in the regulation of gluconeogenesis (e.g., by altering the phosphorylation status of PEPC and PEPCK).

The release of malate from the vacuole will have the following effects [2,97] (Figure 3B). Cytosolic oxaloacetate (OAA) concentration is largely determined by the following reactions (and will increase). Malate and OAA are rapidly interconverted by the reversible enzyme cytosolic MDH (OAA + NADH 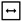 malate + NAD). Further, OAA and 2-oxoglutarate (2-OG) are also interconverted by the reversible reaction catalysed by cytosolic aspartate aminotransferase (AspAT) (glutamate + OAA 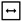 aspartate + 2-OG). Hence an increase in malate concentration will increase OAA concentration (via MDH), and this will then increase aspartate concentration (via AspAT) [126,127,128]. These changes in metabolite concentrations will have a large effect on the in vivo activities of the cytosolic enzymes PEPC, NADP-ME, PEPCK and PK. These enzymes are key regulatory enzymes involved in controlling flux between PEP, pyruvate, malate and OAA [129].

In plants the affinity of PEPCK for OAA is such that physiological increases in OAA concentration will increase greatly flux through the enzyme [2,97,119]. PEPC activity is inhibited by malate, and further its activity is decreased by a fall in pH [137,138,139]. Hence enzyme activity is less at a lower pH, its sensitivity to inhibitory metabolites such as malate is greater, and its sensitivity to activator metabolites is lower [124,129,140]. In CAM plants, the pH of the cytosol decreases when malic acid is released from the vacuole, and this is thought to be important in decreasing flux through PEPC [141]. In addition, the above effects are modulated by the coordinated phosphorylation/dephosphorylation of PEPCK and PEPC. Dephosphorylation increases PEPCK activity, and this arises in part by increasing its affinity for OAA [119]. By contrast, dephosphorylation decreases PEPC activity, and this is brought about in part by increasing its sensitivity to inhibitory metabolites such as malate [129,140]. Thus, in leaves of CAM plants PEPCK and PEPC are dephosphorylated when malate is released from the vacuole, and the reverse occurs when malate is accumulated in the vacuole [2,118]. It is possible (but not certain) that the same protein kinase (PEPC-kinase) is used to phosphorylate PEPCK and PEPC [2,97]. Indeed, PEPC-kinase gene expression is enhanced by an increase in cytosolic pH and PEPCK gene expression is reduced [142]. Further, this change in PEPC-kinase gene expression can lead to rapid changes (less than an hour) in PEPC activity [142,143].

PK converts PEP to pyruvate (PEP + ADP → pyruvate + ATP). Cytosolic PK activity is increased by a decrease in pH, of the order that occurs in the cytosol of leaves of CAM plants when malic acid is released from the vacuole [138,141]. Aspartate concentration is a key factor in determining flux through cytosolic PK in vivo; because aspartate acts as an allosteric activator which over-rides inhibition of the enzyme by glutamate [138,139]. An increase in cytosolic malate concentration will increase cytosolic aspartate concentration (as a result of the reactions of cytosolic MDH and cytosolic AspAT), and hence increase the activity of cytosolic PK.

Cytosolic NADP-ME converts malate to pyruvate (malate + NADP → pyruvate + CO_2_ + NADPH). In the flesh of fruits such as apple, tomato and grape, cytosolic NADP-ME is important in supplying pyruvate to the Krebs cycle [81,89,144,145,146]. Mitochondrial NAD-malic enzyme (NAD-ME) converts malate to pyruvate (malate + NAD → pyruvate + CO_2_ + NADH). This enzyme is important in providing pyruvate for the Krebs cycle, and in Arabidopsis leaves this is associated with the release of malate from the vacuole at night [23]. All forms of ME require a divalent cation to be active [147,148,149,150]. In vitro enzyme assays of ME have employed as the cation either millimolar Mn^2+^ or Mg^2+^, and the catalytic properties depend on the metal ion that is used [148,149,150]. In the cytosol of plant cells, the concentration of Mn^2+^ is submicromolar and that of Mg^2+^ millimolar [121]. Unphysiological concentrations of Mn^2+^ and Mg^2+^ in in vitro assays alter the catalytic properties of many enzymes [121,151]. Studies of cytosolic NADP-ME from grape, and other fruits, have often used unphysiological concentrations of Mn^2+^ in vitro assays, and this has led to conflicting results [152,153]. However, some studies included assays that did not use millimolar concentrations of Mn^2+^ [154], and from these it is clear that the affinity of cytosolic-NADP-ME from grape pericarp for physiological concentrations of malate is sigmoidal at pH 7.3, and that this becomes hyperbolic as the pH drops to around 6.8. A fall in cytosolic pH from 7.3 to 6.8 massively increases the affinity of cytosolic NADP-ME for malate, and this will result in a large increase in the flux through the enzyme. A similar conclusion was reached for both NAD-ME and cytosolic NADP-ME from some other plant tissues [8,148]. Hence an increase in cytosolic malate concentration and a decrease in cytosolic pH will increase flux through PEPCK, cytosolic PK and cytosolic NADP-ME, and this will lead to an increase in pyruvate synthesis.

The Krebs cycle is a large consumer of pyruvate, and its supply must be coordinated with the demand for the products of the cycle (e.g., ATP, NADH and 2-oxoglutarate). In banana flesh an increase in ATP/ADP could be a factor that contributes to reducing flux through cytosolic PK in vivo [139]. An increase in cytosolic 2-oxoglutarate will decrease aspartate concentration (by the action of AspAT), and this will decrease cytosolic PK activity. A decrease in aspartate concentration arising from 2-oxoglutarate synthesis is thought to be important in coordinating cytosolic PK activity and flux through the Krebs cycle with amino acid metabolism [138,139]. Changes in the cytosolic ATP/ADP ratio in the range that can occur in plant cells [155] alter the activity of PEPCK in vivo. A higher ATP/ADP ratio increases the affinity of the enzyme for OAA and hence its activity [97,119]. Hence, when supply of pyruvate exceeds demand, the activity of PEPCK is likely to be maintained, whereas, the activity of cytosolic PK is likely to be reduced and this will increase cytosolic PEP concentration. The cytosolic concentration of PEP is a critical regulator of plant glycolysis, and a high concentration of PEP causes a switch to gluconeogenesis [129].

Subsequently when malic acid is no longer being released from the vacuole its metabolism in the cytoplasm will reduce its concentration. In addition, the metabolism of malate, as described above, consumes the protons arising from the release of malic acid from the vacuole [97]. This decrease in malate concentration and increase in cytosolic pH, will inhibit both PEPCK and cytosolic NADP-ME but activate PEPC. The decrease in flux through PEPCK, and an increase in flux through PEPC, will reduce PEP concentration and this will contribute to a switch from gluconeogenesis to glycolysis.

## 6. Conclusions

In plants, gluconeogenesis is of widespread occurrence and is associated with diverse processes. Often, it is associated with the release of Krebs cycle acids and/or certain nitrogenous compounds from the vacuole, and occurs when the breakdown products of these compounds exceed the demands of other processes. Fine control of enzyme activity is likely to make an important contribution to coordinating both malate synthesis-degradation and the utilisation of malate by gluconeogenesis and the Krebs cycle. The compartmentation of Krebs cycle acids/nitrogenous compounds in the vacuole almost certainly plays a key role in maintaining an appropriate cytoplasmic concentration of these compounds.
